# Medication Adherence in Comorbid Hypertension and Diabetes: An Exploratory Descriptive Qualitative Study of Healthcare Providers' Perspectives

**DOI:** 10.1002/hsr2.73168

**Published:** 2026-09-02

**Authors:** Adwoa Oforiwaa Kwakye, Richard Delali Agbeko Djochie, Ama Agyeman‐Dua, Irene A. Kretchy

**Affiliations:** ^1^ Department of Pharmacy Practice and Clinical Pharmacy, School of Pharmacy, College of Health Sciences University of Ghana Legon Ghana; ^2^ Department of Pharmacotherapeutics and Pharmacy Practice, School of Pharmacy and Pharmaceutical Sciences University of Cape Coast Cape Coast Ghana; ^3^ Pentecost Hospital Madina Accra Ghana

**Keywords:** comorbidity, diabetes, healthcare providers, hypertension, medication adherence

## Abstract

**Background and Aims:**

Medication adherence is a critical determinant of clinical outcomes among patients with comorbid hypertension and diabetes mellitus. Despite the availability of effective therapies, adherence remains suboptimal, particularly in low‐ and middle‐income settings where multiple patient, treatment, and health system factors influence medication‐taking behavior. Healthcare providers are uniquely positioned to identify barriers and facilitators to adherence. This study explored healthcare providers' perspectives on medication adherence among patients with comorbid hypertension and diabetes using the World Health Organization multidimensional adherence framework.

**Methods:**

An exploratory descriptive qualitative study was conducted at the Pentecost Hospital, Ghana, between March and May 2025. Fifteen healthcare providers involved in the management of patients with hypertension and diabetes were purposively sampled, including medical officers, nurses, pharmacist, pharmacy technicians, dietitians, physician assistants, and mental health practitioners. Data were collected through semi‐structured in‐depth interviews lasting 30–60 min and analyzed using thematic analysis following Braun and Clarke's six‐step framework.

**Results:**

Among the 15 participants, 53.3% (*n* = 8) were female, 80.0% (*n* = 12) aged 30–39 years, and 73.3% (*n* = 11) had 1–6 years of professional experience. Five major themes were identified: patient‐related, therapy‐related, condition‐related, socio‐economic, and healthcare system‐related factors. Key barriers included forgetfulness, intentional dose modification, symptom‐driven discontinuation, cultural and religious beliefs about medicines, polypharmacy, medication side effects, fear of complications, anxiety, and medication costs. Facilitators included reminder systems, family and carer support, patient education, and multidisciplinary collaborative care. Participants emphasized the importance of integrating mental health services into chronic disease management.

**Conclusion:**

Healthcare providers perceived medication adherence as influenced by interacting patient, therapy, condition, socio‐economic, and healthcare system factors. Interventions integrating patient education, family involvement, mental health support, and multidisciplinary care may support medication adherence and improve long‐term clinical outcomes.

## Summary

- What is known?
  - Medication adherence in patients with comorbid hypertension and diabetes is often suboptimal.
  - Non‐adherence contributes to poor disease control and increased risk of complications.
- What is new?
  - This study provides qualitative, context‐specific evidence using the WHO multidimensional adherence framework, highlighting healthcare providers' perspectives on adherence barriers and facilitators, including the prominent role of psychological factors, cultural beliefs, and health system constraints.
- Clinical implications
  - Effective adherence interventions should be multi‐component, integrating patient education, family involvement, mental health services, and multidisciplinary collaborative care to address the barriers and improve long‐term outcomes in comorbid hypertension and diabetes.

**Abbreviations**
HCP: health care provider
LMICs: low‐ and middle‐income countries
WHO: World Health Organization

## 1 Introduction

Hypertension and diabetes mellitus are among the most prevalent chronic non‐communicable diseases globally and frequently coexist, particularly in low‐ and middle‐income countries (LMICs), where health systems face increasing strain from multimorbidity [1]. This coexistence increases the risk of complications such as cardiovascular disease and renal failure, and this leads to early mortality. Although effective medications are available, poor adherence remains one of the main challenges to optimal disease management [2, 3]. This may be attributed to the accumulated burden of multiple drugs, asymptomatic disease phases, lifestyle changes, and psychosocial stressors [4]. Research indicates that patients with multimorbidity show lower adherence rates than those with single chronic conditions, while intentional and unintentional non‐adherence is commonly reported [5]. Financial constraints, poor access to medicines, sociocultural beliefs, and fragmented healthcare delivery systems exacerbate such challenges in LMIC settings [6].

The World Health Organization (WHO) conceptualizes medication adherence as a multidimensional phenomenon influenced by five interacting domains: patient‐related, therapy‐related, condition‐related, socio‐economic, and healthcare system–related factors [7, 8, 9]. While this framework is widely used, many adherence studies focus narrowly on patient behavior or single domains, often using quantitative approaches. However, much of the existing qualitative literature prioritizes patient perspectives, with comparatively limited attention given to healthcare providers' experiential insights, particularly in the context of cardiometabolic multimorbidity.

Healthcare providers play a critical role in prescribing, counseling, monitoring, and supporting patients' medication‐taking behaviors [10]. Their perspectives offer a systems‐level understanding of adherence that integrates clinical encounters, patient narratives, and structural constraints within the health system. Exploring providers' views is especially important in settings where task‐sharing, multidisciplinary care, and informal coping strategies are common, yet under‐documented. In addition, providers are uniquely positioned to identify both barriers to adherence and contextually appropriate facilitators that may inform intervention development. Although previous work by our research team at the same institution examined psychological distress and medication adherence among patients with comorbid hypertension and diabetes [11], the present study addresses a distinct research question by exploring healthcare providers' perspectives on the broader range of barriers and facilitators influencing medication adherence.

The aim of this study was to explore healthcare providers' perspectives on barriers and facilitators of medication adherence among patients with comorbid hypertension and diabetes, using the World Health Organization's multidimensional adherence model to provide a comprehensive and theoretically grounded understanding of adherence behavior.

## 2 Methods and Materials

### 2.1 Study Design

An exploratory descriptive qualitative design was employed to examine the perspective of healthcare providers who are directly involved in the management of patients living with both hypertension and diabetes. This design was appropriate for capturing the depth, nuance, and contextual realities underlying providers' experiences and interpretations of patient behaviors and health system challenges.

### 2.2 Study Setting

The study was conducted at the Pentecost Hospital in Ghana. The facility offers both general and specialized medical services, such as Obstetrics and Gynecology, Ophthalmology, Endoscopy, Renal Dialysis, and Dental Health. It has a specialized clinic for people living with hypertension and diabetes. Again, patients receive routine counseling and laboratory monitoring, among others, making it an appropriate setting for this research.

### 2.3 Participant Recruitment and Sampling

Healthcare providers were identified through purposive sampling to ensure representation of professional categories involved in the management of hypertension and diabetes. Medical officers, nurses, pharmacists, pharmacy technicians, dietitians, physician assistants, and mental health practitioners comprised the eligible population. Participants who had at least 6 months of experience in managing patients with hypertension and diabetes formed part of the inclusion criteria. Interviews were continued until data saturation was reached, defined as the point at which no new codes, categories, or conceptual insights emerged from the data [12]. Fifteen healthcare providers participated in this research. This sample size is consistent with qualitative research guidance, which indicates that 12–20 participants are generally adequate for studies examining a focused population and phenomenon [13].

### 2.4 Interviewer Characteristics

All qualitative interviews were conducted by two trained members of the research team. The primary interviewer was a female postgraduate student with a bachelor's degree in public health, specializing in behavioral science, with demonstrated experience in qualitative interviews. The second interviewer was a male clinical pharmacist with a Doctor of Pharmacy degree and experience in patient counseling, chronic disease management, and qualitative data collection. Both interviewers were trained in qualitative interviewing before commencing data collection. To limit power imbalances, participants were engaged as professional colleagues, and it was emphasized that the study aimed to understand experiences rather than evaluate practice.

### 2.5 Data Collection

Data were collected between March 2025 and May 2025 through in‐depth semi‐structured interviews conducted in person depending on participant availability. An interview guide was developed based on the WHO adherence model. Key areas explored included providers' observations of adherence behaviors, perceived barriers and facilitators. Interviews were conducted in English and lasted 30–60 min, and were audio‐recorded with participants' written informed consent. All audio recordings were subsequently transcribed verbatim to ensure accurate representation of participants' accounts before thematic analysis. Field notes were taken to capture non‐verbal cues, contextual details, and emerging reflections. Recruitment ceased once thematic saturation was reached.

### 2.6 Data Management and Analysis

Audio recordings were transcribed verbatim and checked for accuracy against the original files. Data were analyzed using thematic analysis following Braun and Clarke's six‐step framework, which allowed for iterative coding, pattern recognition, and theme generation. The analytic process followed the Consolidated Criteria for Reporting Qualitative Research (COREQ) to ensure methodological transparency and depth [14]. Participants did not review interview transcripts or the study findings (member checking was not undertaken). However, the credibility of the findings was enhanced through independent coding by two researchers, resolution of coding discrepancies through discussion, and adherence to the COREQ to ensure methodological rigor and transparency. An initially inductive coding process was undertaken, followed by deductive mapping of emergent themes onto the WHO multidimensional adherence framework. Themes were reviewed in relation to the entire dataset and interpreted to reflect both individual experiences and shared meanings across professional groups. NVivo software (version 12) was used for data management and coding.

## 3 Results

### 3.1 Participant Characteristics

A total of 15 health care providers were interviewed, comprising professional cadres which included nurses, medical officers, dieticians, pharmacists, pharmacy technicians, mental health practitioners, and physician assistants. Participants ranged in age from their twenties to over 50 years, with the majority (*n *= 12, 80%) aged between 30 and 39 years. Most had between 1 and 6 years (*n *= 11, 73.3%) of professional experience managing chronic disease conditions. Table 1 summarizes the demographic characteristics of the participants.

**Table 1:** Demographic characteristics of participants.

Characteristic	Category	Frequency (*n*)	Percentage (%)
Sex	Female	8	53.3
	Male	7	46.7
Age (years)	20–29	2	13.3
	30–39	12	80.0
	50+	1	6.7
Professional role	Nurse	4	26.7
	Medical officer	3	20.0
	Dietitian	2	13.3
	Mental health practitioner	2	13.3
	Pharmacy technician	2	13.3
	Pharmacist	1	6.7
	Physician assistant	1	6.7
Years of experience	1–3 years	7	46.7
	4–6 years	4	26.7
	7–10 years	3	20.0
	> 10 years	1	6.7

*Note:* Percentages are based on the total sample of 15 participants.

### 3.2 Key Themes

**Figure 1:**
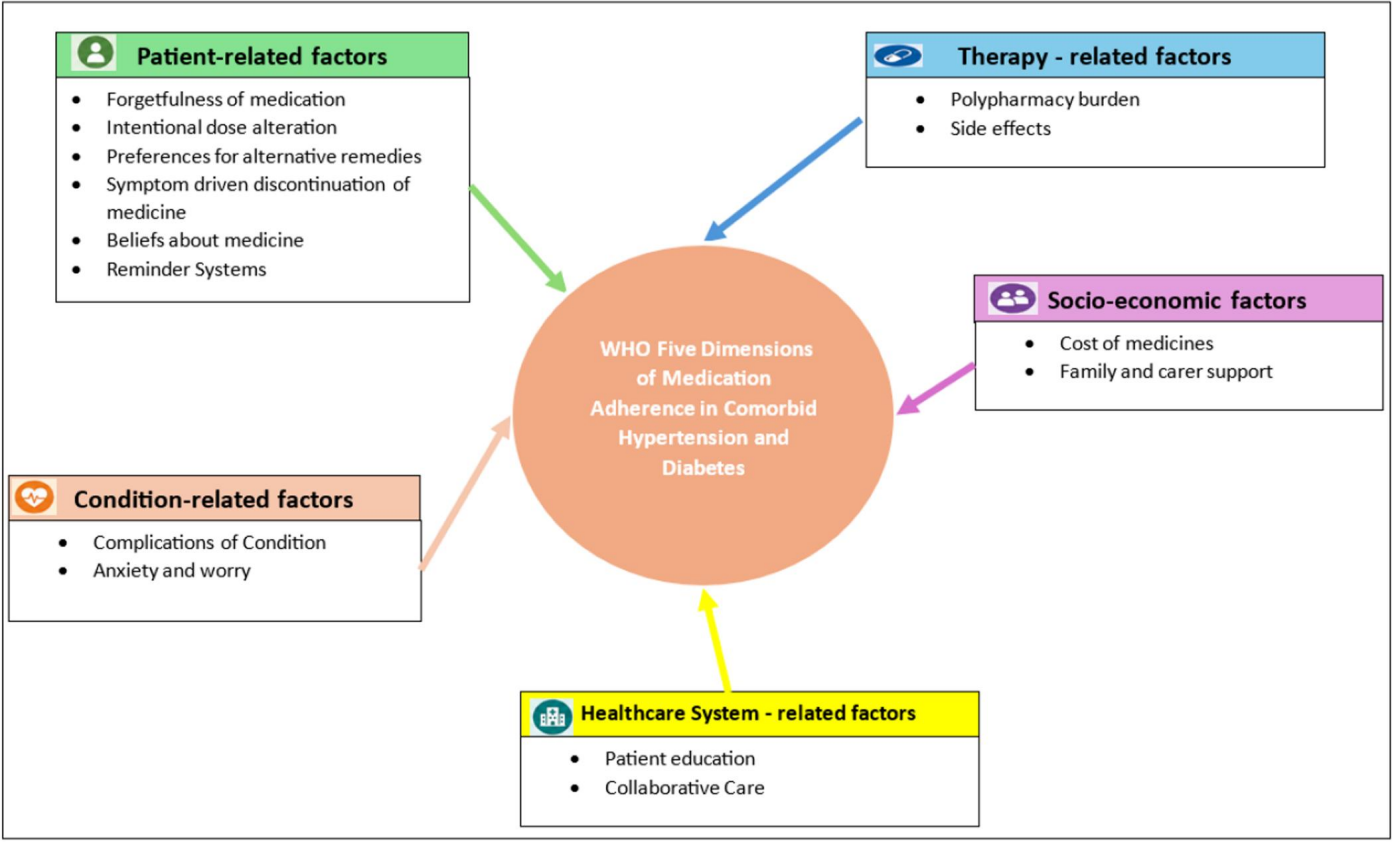
WHO‐aligned thematic framework. WHO‐aligned thematic framework of barriers and facilitators influencing medication adherence among adults with comorbid hypertension and diabetes. The figure presents the thematic framework derived from the perspectives of healthcare providers in the present qualitative study. Findings are organized according to the World Health Organization (WHO) multidimensional framework for medication adherence and grouped into five domains: patient‐related, therapy‐related, condition‐related, socio‐economic, and healthcare system‐related factors.

Five themes were generated based on the WHO multidimensional adherence framework: (1) patient‐related factors, (2) therapy‐related factors, (3) condition‐related factors, (4) socio‐economic factors, and (5) healthcare system‐related factors (see Figure 1). For confidentiality purposes, participants were anonymized and coded as healthcare providers (HCPs) according to the sequence of their responses and age group, for example (HCP 8, 20–29 years). Each theme is presented with relevant subthemes, supported by representative participant quotations that illustrate the experiences and perspectives underpinning the identified themes.

### 3.3 Theme 1: Patient‐ Related Factors

Healthcare providers identified several patient‐related barriers and facilitators that influenced patients' medication adherence.

#### 3.3.1 Forgetfulness of Medication

Healthcare providers frequently identified forgetfulness as a patient‐reported barrier to adherence. One provider explained, *“They will tell you, ‘Oh, I forgot to take it yesterday.’ That is a very common excuse we hear”* (HCP 8, 20–29 years).

#### 3.3.2 Intentional Dose Alteration

Healthcare providers reported that some patients deliberately modify their prescribed regimens, either by skipping doses or prioritizing certain medications, often in response to perceived symptom improvement. One provider noted, *“Some patients will tell you they reduce the number of tablets themselves when they are feeling better”* (HCP 1, 30–38 years), while another added, *“They change their regimen without informing us, sometimes taking only the blood pressure drugs and leaving the diabetes ones”* (HCP 2, 20–29 years).

#### 3.3.3 Preference for Alternative and Traditional Remedies

Healthcare providers reported that some patients prefer herbal or faith‐based treatments over prescribed medications, particularly after hospital discharge. As one provider explained, “Most are now more into herbal medicines. When they're in the hospital, they prefer to use the orthodox medicine; once they are discharged, they prefer to take in herbal medications” (HCP 15, 20–29 years). Another practitioner added, “…They may say “*oh these herbs can help you better than the orthodox medication… because of our belief system, they believe what the pastor and the herbalist is saying more than what the doctor, the expert is saying”* (HCP 7, 30–39 years).

#### 3.3.4 Symptom Driven Discontinuation of Medicine

Healthcare providers reported that some patients intentionally stop taking medications once they experience symptom relief, often leading to relapse. One provider noted, “*Some think once they feel better, they can stop, so they relapse”* (HCP 2, 20–29 years).

#### 3.3.5 Beliefs About Medicine

Healthcare providers reported that patients' beliefs about medicines, shaped by religious and cultural influences, affect adherence to prescribed therapy. As one mental health practitioner explained, *“…Number one is religion, culture. These are the two main reasons… there're a lot of false teaching going on that ‘come let me pray for you and your sickness will go’”* (HCP 5, 30–39 years).

#### 3.3.6 Reminder Systems

Healthcare providers reported that practical reminder strategies support adherence. One provider explained, *“Some use pill boxes to organize for the week, and it helps them”* (HCP 9, 30–39 years).

### 3.4 Theme 2: Therapy‐Related Factors

Healthcare providers reported that treatment burden and regimen‐related factors contributed to patients' medication adherence.

#### 3.4.1 Polypharmacy Burden

Healthcare providers highlighted the challenges of managing multiple medications, noting that patients often become fatigued when prescribed numerous drugs. One provider explained, “Some patients are on more than seven medicines… they get tired” (HCP 10, 30–39 years).

#### 3.4.2 Side Effects

Healthcare providers reported that adverse effects from medications contribute to non‐adherence, with patients sometimes discontinuing therapy in response to bothersome symptoms. As one provider explained, *“They complain that when they take it, they urinate too much, so they stop”* (HCP 10, 30–39 years).

### 3.5 Theme 3: Condition‐Related Factors

Healthcare providers reported that condition‐related challenges and disease burden influenced patients' medication adherence.

#### 3.5.1 Complications of Condition

Healthcare providers reported that patients' fear of serious complications, such as stroke and kidney failure, affects their emotional wellbeing and engagement with treatment. As one practitioner explained, “They always talk about stroke and kidney failure; you can see they have lost hope” (HCP 15, 20–29 years).

#### 3.5.2 Anxiety and Worry

Healthcare providers observed that patients frequently experience anxiety and worry about their long‐term treatment and health outcomes. One provider explained, *“Most of them are anxious… they always ask, ‘Will I ever stop taking this medicine?’… Sometimes we have patients crying and all that. So it's quite common, but usually after the first conversation, when they start to see results as well, that side improves”* (HCP 4, 30–39 years).

### 3.6 Theme 4: Socio‐Economic Factors

Healthcare providers reported that financial constraints and social factors influenced patients' medication adherence.

#### 3.6.1 Cost of Medicines

Healthcare providers identified medication costs as a significant barrier to adherence, often creating financial strain that competes with basic needs. As one provider noted, “*They feel stressed because after buying medicine, nothing is left for food”* (HCP 10, 30–39 years).

#### 3.6.2 Family and Carer Support

Healthcare providers emphasized the positive role of family and carers in supporting medication adherence, particularly when relatives living with patients are actively involved in care. As one provider noted, “*When you involve the family, adherence is always better”* (HCP 13, 30–34 years). Similarly, another provider highlighted the need for inclusive counseling, stating, *“I think while we talk to the clients, we also need to be talking to their relatives who are living with them in the house”* (HCP 12, 20–29 years).

### 3.7 Theme 5: Healthcare System‐Related Factors

Healthcare providers reported that health system–related facilitators influenced patients' medication adherence.

#### 3.7.1 Patient Education

Healthcare providers identified patient education as a key health system–related facilitator of medication adherence, emphasizing that improved understanding of disease and treatment promotes behavioral change. As one provider stated, “*Education, if we can let them know what it's all about, the real truth… when people really understand, things change”* (HCP 14, 30–39 years).

#### 3.7.2 Collaborative Care

Healthcare providers emphasized the importance of multidisciplinary collaboration in supporting medication adherence, particularly the integration of mental health services into routine chronic disease management. As one provider noted, *“The mental health unit and a trained counsellor should be a part of our team management”* (HCP 3, 30–39 years).

## 4 Discussion

Consistent with the World Health Organization's multidimensional adherence framework, the findings demonstrate that medication adherence is shaped by an interplay of patient‐related, therapy‐related, condition‐related, socio‐economic, and healthcare system factors [7]. Importantly, the providers' narratives highlight not only barriers but also practical facilitators, offering contextually relevant implications for intervention design in low‐ and middle‐income settings.

At the patient level, forgetfulness emerged as a common barrier, with unintentional non‐adherence remaining prevalent among individuals managing chronic conditions [15]. Furthermore, forgetfulness may reflect deeper challenges such as cognitive overload, competing life demands, or insufficient integration of medication‐taking into daily routines [16]. However, the reported use of reminder systems, including pill boxes, aligns with evidence supporting simple adherence aids as effective, low‐cost strategies, particularly in resource‐constrained contexts [17]. Intentional dose alteration and symptom‐driven discontinuation were also frequently reported. Observations by providers of patients reducing doses or selectively prioritizing antihypertensive over antidiabetic medications highlight patients' focus on conditions perceived as more immediately threatening or symptomatic [18]. Furthermore, beliefs about medicines were highlighted in providers' narration. A preference for herbal and faith‐based remedies, particularly post‐discharge, is well‐documented in sub‐Saharan Africa and reflects the sociocultural belief systems coupled with a lack of trust in biomedical therapies [19]. Again, providers also identified misinformation provided by religious leaders and herbal practitioners as a major driver of nonadherence. This reiterates calls for community‐level interventions that engage faith leaders as partners in disease management [20].

Furthermore, therapy‐related factors, particularly polypharmacy and side effects, were described as contributors to nonadherence. This is consistent with prior studies showing that increasing pill burden is inversely associated with adherence in patients with comorbid hypertension and diabetes [21]. Side effects such as frequent urination, which is associated with some of the antihypertensive therapy, were reported to have been the cause of medication discontinuation, highlighting the importance of patient counseling [22].

Condition‐related psychological factors such as fear of complications, anxiety, and emotional distress were salient in providers' narratives. This is consistent with findings of high prevalence of anxiety and depressive symptoms among patients with cardiometabolic conditions [23, 24]. These findings strengthen the argument for integrating mental health screening and support into routine hypertension and diabetes care.

Socio‐economic constraints, particularly medication cost, were found to be a barrier to adherence. The decision between having to buy medicines and meeting basic needs reiterates findings from other low‐resource settings where out‐of‐pocket expenditure remains a key driver of non‐adherence despite insurance schemes [25, 26]. Family and carer support emerged as a facilitator of adherence, which aligns with evidence that social support promotes adherence through reminders and providing emotional encouragement [27].

At the health system level, patient education and collaborative care were highlighted as critical facilitators. Notably, the call for integrating mental health professionals into chronic disease teams resonates with the collaborative care model, which has demonstrated effectiveness in improving both psychological outcomes and medication adherence in patients with diabetes and cardiovascular disease [28].

## 5 Strengths and Limitations

The qualitative design allowed for deep and nuanced insights into contextual, cultural, and health system dynamics that quantitative approaches may overlook. The inclusion of participants from multiple professional disciplines, including medical officers, nurses, pharmacists, pharmacy technicians, dietitians, physician assistants, and mental health practitioners, provided diverse perspectives and strengthened the multidisciplinary understanding of chronic disease management and medication adherence. However, the study was conducted at a single healthcare facility and involved purposively selected healthcare providers. Although purposive sampling enabled the recruitment of participants with direct experience in managing patients with comorbid hypertension and diabetes, it may have introduced selection bias, as the perspectives of participants who were not recruited or who chose not to participate may differ. Furthermore, the experiences and views of healthcare providers at one facility may not adequately capture variations in chronic disease management practices, resources, organizational structures, and patient populations across other regions or levels of the Ghanaian health system. Therefore, the findings should not be interpreted as statistically generalizable to all healthcare providers or healthcare settings. Rather, the findings offer context‐specific insights, and their transferability to other settings should be considered in relation to similarities in healthcare organization, professional roles, patient populations, and available resources.

## 6 Conclusion

This research indicates that medication adherence in patients with comorbid hypertension and diabetes is influenced by interrelated patient, therapy, psychological, socio‐economic, and health system factors. Forgetfulness, intentional dose adjustment, cultural and religious beliefs, treatment burden, psychological distress, and medication costs were the main barriers, whereas family support, reminder systems, and patient education facilitated adherence. Healthcare providers stressed the need for collaborative care with particular integration of mental health services into chronic disease management. Strategies targeting these factors are required through patient‐centered, contextually adapted interventions if improved sustained adherence and long‐term clinical outcomes are to be achieved in resource‐constrained settings.

## Author Contributions

**Adwoa Oforiwaa Kwakye:** conceptualization, investigation, funding acquisition, writing – original draft, methodology, validation, visualization, writing – review and editing, supervision, project administration. **Richard Delali Agbeko Djochie:** investigation, writing – original draft, methodology, validation, visualization, writing – review and editing, formal analysis, data curation, software. **Ama Agyeman‐Dua:** methodology, validation, visualization, writing – original draft, writing – review and editing, data curation, resources. **Irene A. Kretchy:** conceptualization, methodology, validation, visualization, supervision, writing – review and editing, writing – original draft.

## Ethics Statement

Ethical approval was obtained from the Christian Health Association of Ghana (CHAG) Research Department Institutional Review Board (Ref: CHAG‐IRB/020/2024). Administrative permission was granted by the participating facility. All participants received detailed information about the study aims, procedures, confidentiality measures, and their right to withdraw at any time without consequence. Written informed consent was obtained from all participants prior to data collection. Any identifiable information was removed from transcripts, and all data were secured on password‐protected systems that only the research team had access to.

## Conflicts of Interest

The authors declare no conflicts of interest.

## Transparent Statement

Adwoa Oforiwaa Kwakye affirms that this manuscript is an honest, accurate, and transparent account of the study being reported; that no important aspects of the study have been omitted; and that any discrepancies from the study as planned (and, if relevant, registered) have been explained.

## Data Availability

All relevant data are presented within the manuscript. De‐identified qualitative data generated and/or analyzed during this study, including relevant interview transcript excerpts, may be made available from the corresponding author upon reasonable request, subject to appropriate confidentiality safeguards. Raw audio recordings and transcripts containing potentially identifiable information will not be publicly available because of the risk of participant identification.
